# Parent attitudes towards data sharing in developmental science

**DOI:** 10.12688/openreseurope.16516.1

**Published:** 2023-10-20

**Authors:** Jannath Begum Ali, Rebecca Holman, Amy L. Goodwin, Siofra Heraty, Emily J.H. Jones

**Affiliations:** 1 Department of Psychological Sciences, Birkbeck University of London, London, England, UK; 2 Department of Forensic and Neurodevelopmental Sciences, King's College London, London, England, UK

**Keywords:** Open science, data sharing, typical development, Autism spectrum disorder, neurodevelopmental conditions, developmental science, consent

## Abstract

**Background:**

Data sharing in developmental science is increasingly encouraged, supported by funder and publisher mandates for open data access. Data sharing can accelerate discovery, link researchers with high quality analytic expertise to researchers with large datasets and democratise the research landscape to enable researchers with limited funding to access large sample sizes. However, there are also significant privacy and security concerns, in addition to conceptual and ethical considerations. These are particularly acute for developmental science, where child participants cannot consent themselves. As we move forward into a new era of data openness, it is essential that we adequately represent the views of stakeholder communities in designing data sharing efforts.

**Methods:**

We conducted a comprehensive survey of the opinions of 195 parents on data sharing in developmental science. Survey themes included how widely parents are willing to share their child’s data, which type of organisations they would share the data with and the type of consent they would be comfortable providing.

**Results:**

Results showed that parents were generally supportive of curated, but not open, data sharing. In addition to individual privacy and security concerns, more altruistic considerations around the purpose of research were important. Parents overwhelmingly supported nuanced consenting models in which preferences for particular types of data sharing could be changed over time. This model is different to that implemented in the vast majority of developmental science research and is contrary to many funder or publisher mandates.

**Conclusions:**

The field should look to create shared repositories that implement features such as dynamic consent and mechanisms for curated sharing that allow consideration of the scientific questions addressed. Better communication and outreach are required to build trust in data sharing, and advanced analytic methods will be required to understand the impact of selective sharing on reproducibility and representativeness of research datasets.

## Introduction

Developmental cognitive neuroscience is a burgeoning field ( Nketia *et al.*, 2021). Understanding the brain and cognitive changes that underpin the dramatic changes in behaviour over the first years of life is critical to a fundamental etiological understanding of human functioning. Studying brain development is also central to robustly testing the assumptions of theoretical models of brain function constructed to explain adult data. Finally, many neurodevelopmental conditions are highly heritable and connected to genes with peak periods of expression prenatally, thus likely impacting early brain development ( Ismail *et al.*, 2017). Therefore, developing a robust science of early brain and cognitive development that can support inferences about both general development and individual differences is critical. There is mounting evidence that robust science requires data sharing, because this allows assessment of reproducibility ( Gilmore & Qian, 2022), generalisability to different analytic approaches (e.g., Poldrack *et al.*, 2013), diversification of samples (see Hindorff *et al.*, 2018 for a review) and increased power ( Jones *et al.*, 2019). However, efforts to share paediatric data are complicated by the ethical considerations around sharing data from participants who cannot themselves consent. As we move forward with data sharing efforts, it is important to consider the views of parents and families on the design and governance of data sharing endeavours to ensure that data is shared responsibly.

 Broader debates around data sharing are becoming increasingly heated. On the one hand, advocates of open science rightly highlight the potential benefits of open data sharing (e.g., Milham *et al.*, 2018). These include improving reproducibility, which is as substantial a challenge in developmental science ( Frank *et al.*, 2017) as in many other fields; helping with the democratisation of research data, ensuring access to scientists in historically under-resourced settings ( Kobayashi *et al.*, 2021); improving statistical power and the representativeness of cohorts by enabling larger sample sizes through data pooling and shared protocols ( Jones *et al.*, 2019); and allowing other researchers to probe the generalisability of findings across variation in analysis pipelines or data cleaning approaches ( Carp, 2012; Poldrack & Gorgolewski, 2014). These laudable goals have led to many funders adopting mandates for data sharing. Journals may mandate statements about data accessibility and encourage placement of data in public repositories, with ‘badges’ awarded for compliance ( Kidwell *et al.*, 2016). These efforts are important, but there are also real concerns around the culture of ‘bropen science’ ( Whitaker & Guest, 2020), whereby only a narrow demographic of researchers are able to benefit from open science practices. In particular, some consider that aspects of the open science movement have become unnecessarily dogmatic and hegemonic (e.g., McDermott, 2022). Further, there has been little focus on the gender distribution of researchers who exploit open datasets (sometimes called ‘data parasites’; Longo & Drazen, 2016) relative to those who often are involved in preparing them. This can have real implications for the career development of researchers who collect and share data (particularly if they are at an earlier career stage) because this process requires a considerable investment of time relative to running analyses on existing data. Further, the needs and wishes of participants are often not the primary focus of many efforts to improve transparency and visibility, in part because such efforts are often driven by researchers who are not working directly with the communities from whom data is collected.

Concurrent with these efforts, there has increasing concern about privacy, security and the values of those accessing research data. The introduction of GDPR legislation in Europe has highlighted the need to provide a purpose for which personal information is shared or collected; in the context of research, any data linked to an individual through an ID is considered to be personal information. There is meaningful concern that GDPR is hampering sharing of data for research or medical purposes far more than the use of data by companies that it was primarily designed to restrict ( Vukovic *et al.*, 2022). Widely publicised scandals such as the misuse of Facebook data by Cambridge Analytica ( Berghel, 2018) and identifiable health care data used by Google (the Nightingale Project; Ledford, 2019) have raised further questions of trust in industry and have highlighted potential risks of data sharing. Finally, the neurodiversity movement has increased the profile of concerns around the misuse of data on individuals with neurodevelopmental conditions, with a particular focus on genetics and the long shadow of eugenics ( Sanderson, 2021).

Developmental cognitive neuroscience has been largely insulated from these debates to date, with most focus on large-scale adult datasets, biobanks or specific clinical populations. However, there are increasing efforts to generate large-scale developmental brain and cognitive data that include consortia focused on basic science ( Frank *et al.*, 2017); the early development of cohorts of infants enriched for neurodevelopmental conditions ( Jones *et al.*, 2019; Volkow *et al.*, 2021); and the inclusion of biological measures into ongoing population studies ( Magnus *et al.*, 2006; Magnus *et al.*, 2016). As data sharing possibilities evolve with the advent of new technologies, there is pressing need to consider the views of parents on the collection and sharing of their child’s data. This is particularly critical as sharing mandates become more common at developmental journals (e.g., Gennetian *et al.*, 2022). The previous literature in this area has typically focused on genetic sharing (e.g. Yamamoto *et al.*, 2021), and general biobanking ( Antommaria *et al.*, 2018; Halverson & Ross, 2012). For example, previous interview or questionnaire studies have shown that parents are generally supportive of data sharing, but with concerns around privacy, security and shared values between themselves and experimenters ( Manhas *et al.*, 2015). For example, an interview study with 19 interviewees and 18 focus-group participants selected from participants in an existing birth cohort study found generally strong support for data sharing ( Manhas *et al.*, 2016). However, the study found that altruism has limits. Participants had remaining concerns about privacy and security and some areas of divergence in opinion, including on sharing data with industry and the nature and composition of data access panels. Although one study indicated support for broad consent for data sharing ( Manhas *et al.*, 2016), another indicated that families were much more likely to refuse sharing for their child’s biological data than their own ( Burstein *et al.*, 2014) and would select restricted sharing options if given the choice ( Burstein *et al.*, 2014). In a survey of families asked to think about biobanking data for children who were sick, the nature of a disease affecting their child would influence parent views on biobanking ( Salvaterra *et al.*, 2014). Thus, the existing literature suggests that families do have concerns about data sharing but recognise its power and potential.

Taken together, the majority of focus in the literature has been biobanks (in which samples are taken specifically for long-term storage) and genetic studies. Less is known about parental views on sharing other modalities commonly used in developmental science and collected for a specific research purpose and later shared (for example, including such measures such as imaging, eyetracking, electroencephalography and cortisol levels). Further, although some work shows that the presence of a medical condition might impact sharing ( Salvaterra *et al.*, 2014), no studies have considered whether decision-making is influenced by having a family history of neurodevelopmental conditions. This is timely to explore because many recent concerns have emerged as part of the neurodiversity movement ( Hobson *et al.*, 2022). Finally, few studies have explored the factors that influence sharing such as geographical location and nature of the receiving partner (though a range of studies have shown that trust in industry can be low; e.g., Manhas *et al.*, 2015).

To fill these gaps, we conducted a comprehensive survey of 195 parents with and without a family member with a neurodevelopmental condition and with a child under 18 years. Most families had some level of previous research participation (c.94.4%), and thus were generally familiar with research practices. We focused on three broad questions: ***Who can share the data?*** We examined participants' willingness to share data with different types of organisations; across different geographical locations; and the factors that most influenced their decisions. ***How is the data shared?*** We asked participants about their views of common consenting models, including Restrictive, Dynamic, Tiered and Broad consent. ***What data is shared*?** We asked whether parents think differently about sharing data such as brain scans compared to sharing video recordings of behavioural tasks. We examined whether responses differed between families with and without a family history of neurodevelopmental conditions.

## Methods

### Study design

Participants completed an online questionnaire examining their attitudes towards sharing their child’s data that had been collected within a research setting. The questionnaire was live between 2020 to 2021.

### Recruitment procedure

Participants were recruited via the Birkbeck Babylab. Parents registered on the database were emailed an online questionnaire link (see *Extended data* ( Begum, 2023)). As part of signing up to the database, participants had previously consented to be contacted for future research. In addition to this, we also advertised the questionnaire via social media platforms (e.g., Twitter, Facebook and Instagram). Both recruitment methods require participants to click on the study weblink which redirects to the participant information sheet (see *Extended data* ( Begum, 2023)), as such participants were recruited on a voluntary basis. Recruitment continued until the questionnaire link had been live for a year. Consent was gathered via tick box; participants had to complete the online consent before being able to move onto the questionnaire. Inclusion criteria for the study was based on two factors; being a parent or legal guardian of a child under the age of 16 and currently being based in the UK. Participants were not rewarded with any compensation, monetary or otherwise, for taking part in the study. Ethical approval was provided by Birkbeck University of London Research Ethics Committee (ID: 192092)

### Questionnaire

The survey was designed in Gorilla by researchers at the Centre for Brain and Cognitive Development, Birkbeck University of London for the purpose of this study, consisting of 57 questions, split into four sections (see *Extended data* ( Begum, 2023)). The questionnaire took an average of 25 minutes for parents to complete. Parents were asked throughout the study to answer each question with their youngest child ( *mean age*= 59.44 months, *SD* = 45.11 months) in mind unless the question specifically stated otherwise. Section 1 was designed to collect basic demographic information about their youngest child (sex, age, ethnicity) and family (parent education, presence of neurodevelopmental or genetic conditions). Sections 2 to 4 asked participants about the types of organisations that parents would be willing to share data with (either their own or their child’s), the level of control they would want over the data sharing process, what might influence their decision to share data and what type/form of data they would be willing to share. The survey consists of open-ended and closed questions. A small pilot group provided feedback on an earlier version of the survey (n=5).

### Participants

**Figure 1.**
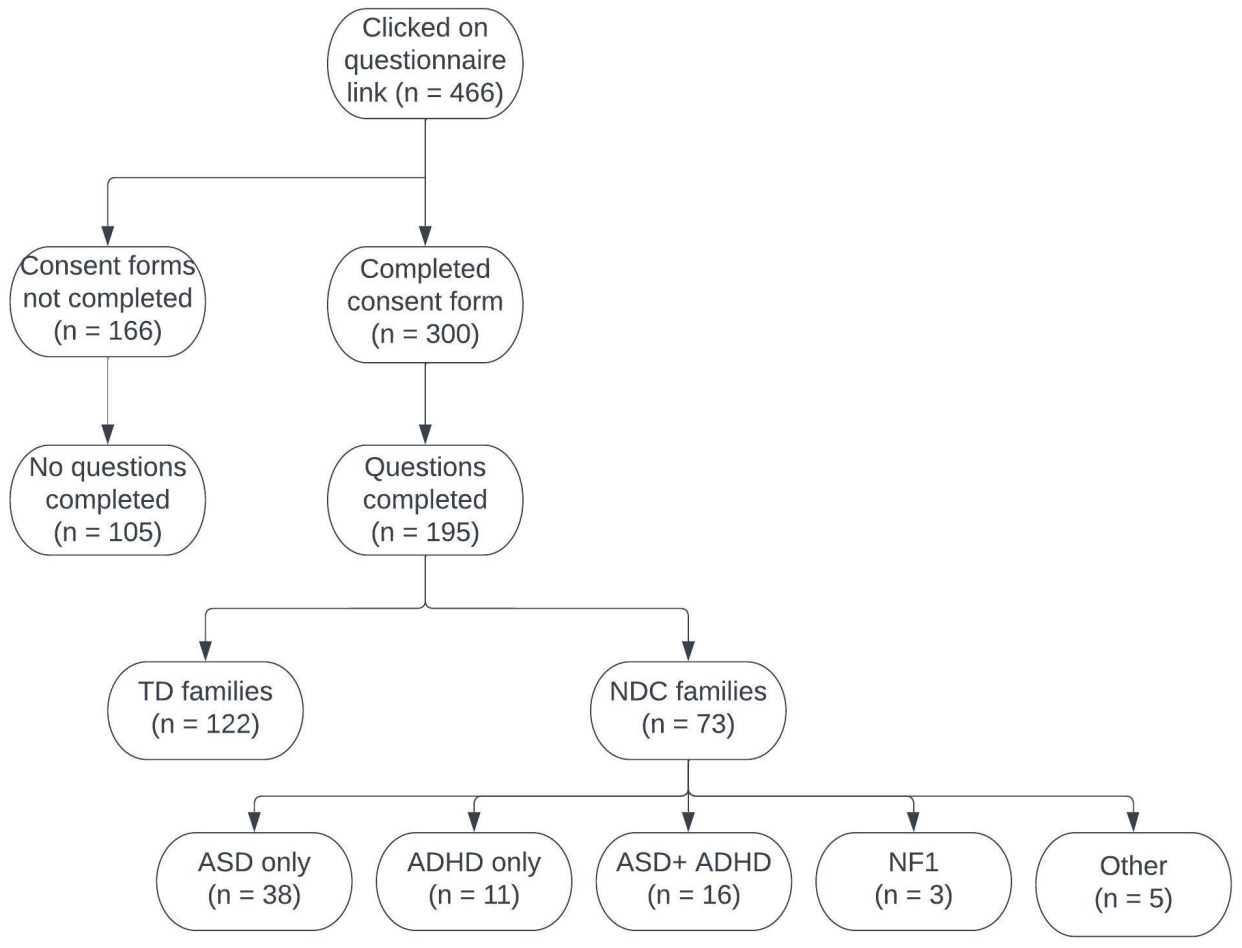
Consort diagram showing the total number of participants in our sample.

466 individuals clicked on the study weblink, 166 of these did not complete the consent form and therefore did not get access to the study. A further 105 individuals completed the consent form but did not answer any questions, both these groups were not included in the analysis. 195 participants are included in the study (see Figure 1); 183 mothers and 12 fathers. Of the 195 respondents, 122 had typically developing (TD) children and reported no neurodevelopmental conditions in the target child, siblings or parents, while 73 families reported at least one neurodevelopmental condition (NDC) in the immediate family (38 ASD, 11 ADHD, 16 ASD+ADHD, 3 NF1, 5 Dyslexia/dyspraxia/Epilepsy); see Table 1 for sample demographics.

**Table 1.** Sample demographics.

	Total Sample n (%)	TD n (%)	NDC n (%)
**Child’s Sex**			
Female	96 (49.2)	60 (49.2)	36 (49.3)
Male	99 (50.8)	62 (50.8)	37 (50.7)
**Child’s Ethnicity**			
White	145 (74.4)	85 (69.7)	60 (82.2)
Black	5 (2.6)	3 (2.5)	2 (2.7)
Asian	11 (5.6)	8 (6.6)	3 (4.1)
Mixed race/Other	33 (16.9)	25 (20.5)	8 (11.0)
Prefer not to answer	1 (0.5)	1 (0.8)	0
**Parental Education**			
Primary	2 (1)	2 (1.6)	0
Secondary	22 (11.3)	5 (4.1)	17 (23.3)
Tertiary- Undergraduate	61 (31.3)	39 (32.0)	22 (30.1)
Tertiary- Postgraduate	108 (55.4)	74 (60.7)	34 (46.6)
Prefer not to answer	2 (1)	2 (1.6)	0

### Statistical analysis plan

Statistical analysis was conducted using SPSS (IMB version 26.0.0.0). Non-parametric tests were used due to the ordinal and categorical nature of the data. Answers to open questions were explored using Leximancer Desktop 5.0; this was not intended to be an exhaustive analysis but instead to generate concepts and themes which may be important to consider or highlight other factors not yet explored by the survey. The Leximancer’s concept tool was used to display the prevalence and co-occurrence of generated themes/concepts within the text.

## Results

### Who can share my child’s data?

Participants were asked to select the types of organisations with whom they would be happy to share their child’s data. This showed strong differences in views by sector: of the total sample 96.4% would be happy to share their child’s data with universities and research centres, while only 16.9% of participants would be happy to share their child's data with private companies and industry. Families with a history of neurodevelopmental conditions (NDC) were more willing than families without a history of NDC to share their child’s data with GPs and hospitals (NDC=95.9% vs TD=85.2%, X ^2^(1)=5.38, *p*=.02, V=.17), private companies and industry (NDC=24% vs TD =12%, X ^2^(1)=4.97, *p*=.026, V=.16) and charities (NDC=58% vs TD=31%; X ^2^(1)=13.14, *p*<.001, V=.26). The two groups did not differ significantly in their willingness to share their data with universities and research centres (NDC=99% vs TD=95%; X ^2^(1)=1.66, *p*=.197, V=.1).

Parental education did not impact the type of organisation parents were willing to share their child’s data with [GPs and hospitals: (X ^2^(2)= .189, *p*= .910, V= .031; universities and research centres: (X ^2^(2)= 1.196, *p*= .550, V= 0.79; private companies and industry: (X ^2^(2)= .383, *p*= .826, V= .045 and charities: (X ^2^(2)= .669, *p*= .716, V= .059; Table S3], nor was this affected by parental ethnicity [GPs and hospitals: (X ^2^(3)= 2.080, *p*= .556, V= .104; universities and research centres: X ^2^(3)= 1.471, *p*= .689, V= 0.87; private companies and industry: (X ^2^(3)= 1.399, *p*= .706, V= .085 and charities (X ^2^(3)= 3.435, *p*= .329, V= .133; Table S4].

***Trust in each type of organisation.*** Participants were asked to rate their level of trust in these four types of organisations on a Likert scale from 1(Do not trust) to 5 (Trust completely). Friedman's analysis of variance was conducted with Dunn’s pairwise comparisons and Bonferroni correction for multiple comparisons (alpha corrected to p = .008). Participant-reported trust in sharing their child's data significantly differed depending on the type of organisation considered; GPs/hospitals ( *M*=3.90, *SD*=0.87) universities/research centres ( *M*=4.20, *SD*=0.71), private companies/industries ( *M*=2.10, *SD*=1.12) and charities ( *M*=2.90, *SD*=1.15), χ ^2^F(3)=397.63, *p*<.001, W=.68). Dunn’s post-hoc tests with Bonferroni adjustments revealed participants significantly rated private companies and industries less trustworthy than charities (z=-.76, *p*<.001), GPs and hospitals (z=1.79, *p*<.001), and universities/research centres (z=2, *p*<.001). Charities were also rated significantly less trustworthy than GPs and hospitals (z=1.03, *p*<.001) and universities and research centres (z=1.24, *p*<.001). 

**Figure 2.**
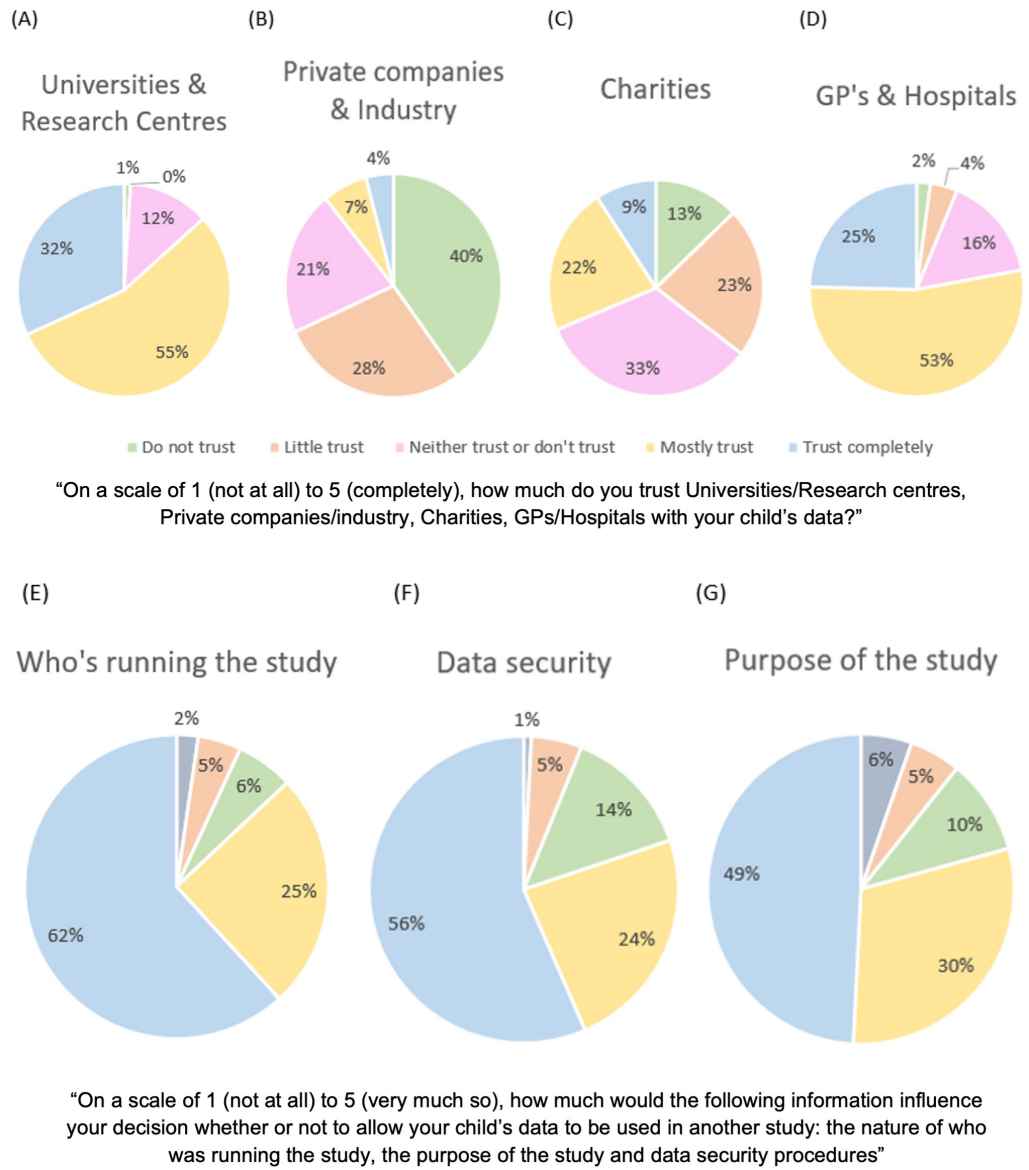
Graphs showing participants’ trust levels with different organisations (Panel **A**: Universities and Research Centre’s, Panel **B**: Private Companies and Industry, Panel **C**: Charities, Panel **D**: GP’s and Hospitals), that may share their data. Graphs showing which factors influence parents decisions to share data (Panel E: Who is running the study, Panel **F**: Data security procedures, Panel **G**: Purpose of the study).

Trust in organisations did not differ by parental education level [GPs/hospitals; X ^2^(2)=3.91, *p=*.141, η2=.02, universities/research centres; X ^2^(2)=5.13, *p=*.08, η2=.03, private companies; X ^2^(2) = 3.66, *p=*.16, η2 = .02, charities; X ^2^(2)=1.24, *p=*.55, η2=.006]. However, parents with children with NDCs trusted private companies more than parents with only TD children ( *U*=5193, *z*=2.04, *p*=.04, η2=.02) (mean rank TD=91.93, NDC=108.14). Our NDC group also trusted charities more than the TD group ( *U*=5246, *z*=2.15, *p*=.03, η2=.024) (mean rank TD=91.5, NDC=108.86). We found no significant differences in parental trust of GPs/hospitals ( *U*=4663, *z*=.61, *p*=.55, mean rank TD=96.28, NDC=100.88) or universities/research centres ( *U*=4531, *z*=.23, *p*=.82; mean rank TD=97.36, NDC=99.07) between our NDC and TD groups; see Figure 2.

Trust in organisations also did not differ by parental ethnicity [GPs/hospitals; X ^2^(3)=1.575, *p=*.665, η2=.004, Uuniversities/research centres; X ^2^(3)=.606, *p=*.895, η2=.002, private companies; X ^2^(3) = 4.073, *p=*.254, η2 = .022, charities; X ^2^(3)=1.098, *p=*.778, η2=.005; Table S5.]

**Figure 3.**
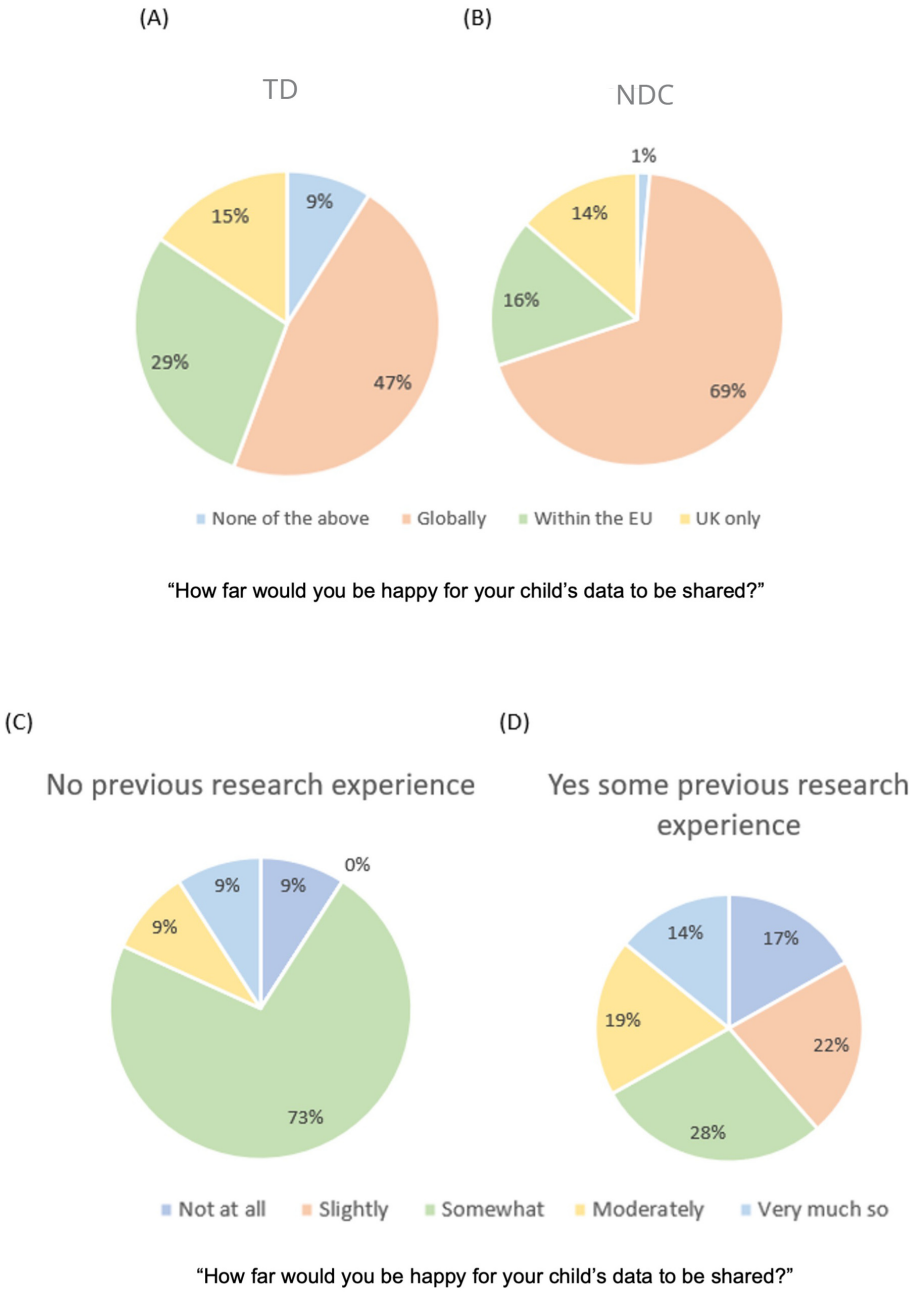
Graphs showing participants preference in distance of sharing data, split by group (Panel **A**: Families without a child with a neurodevelopmental condition, Panel **B**: Families with a child with a neurodevelopmental condition). Graphs showing participants’ level of worry about sharing data, split by whether they had any previous experience of taking part in research (Panel **C**: No previous research experience, Panel **D**: Some research experience either parent or child).

***Reach of sharing.*** When asked how far they would be happy for their child's data to be shared, the majority of participants (54.9%) responded ‘Globally’, followed by ‘Within the EU’ (24.1%), ‘UK only’ (14.9%) and ‘None of the above – data to be shared only with the researchers of the original study’ (6.2%). Neither parental education [Kruskal-Wallis: *H*(2)=1.81, *p=*.4, η2=.001] nor parental ethnicity [Kruskal-Wallis: *H*(3)=2.06, *p=*.56, η2=.005] affected how widely parents were willing to share their child’s data. We found a significant difference between TD and NDC parents on how widely they would share their child’s data [X ^2^(3)=11.236, *p*=.01, V=.240]. Follow-up tests using adjusted z-values and Bonferroni correction for multiple comparisons (alpha corrected to *p*=.006) found that NDC families were more willing to share their child’s data globally X ^2^(1)=8.76, *p*=.003 (68.5% of NDC families vs 46.7% of TD families). There were no group differences in parents’ willingness to share their child’s data within the EU [NDC=16.4%, TD=28.7%, X ^2^(1)=3.76, *p*=.05], keep the data within the UK [NDC=13.7%, TD=15.6%, X ^2^(1)=0.13, *p*=.72] or those that chose ‘None of the above’ [NDC=1.4%, TD=9%, X ^2^(1)=4.62, *p*=.03]; see Figure 3.

***Influences on parent’s decisions to share child data.*** To try and distinguish if the type of organisation was the main factor influencing parental decision making, participants were asked to rate three possible factors which may influence their decision to share their child’s data: who is running the study, the purpose of the study and the data security procedures of the study, using a Likert scale from 1 (Not at all) to 5 (Very much so). Friedman's analysis of variance found a significant difference in how important participants rated the three factors, [χ ^2^(2)=6, *p*=.05, W=.023]. The organisation running the study ( *M*=4.4, *SD*=0.96), data security procedures ( *M*=4.3, *SD*=0.95) and the purpose of the study ( *M*=4.1, *SD*=1.13) were all important factors; post-hoc tests with Dunn’s pairwise comparisons (Bonferroni corrected to p<.001 for multiple comparisons) found that the purpose of the study was not rated significantly lower than data security (z=-.081, *p*=1) or who the study was run by (z=.2, *p*=.34). Data security was not rated significantly differently to who was running the study (z=.12, *p*=1); see Figure 3. Thus, the purpose for which data is shared is equally important to families as the security with which it is shared, and who data is shared with.

Mann-Whitney U tests were used to investigate if there were differences between the NDC and TD groups on the three factors that may influence a parent's decision to share their child's data. NDC families reported that the purpose of the study is more important to them ( *M*=4.6, *SD*=.76) than TD groups ( *M*=3.89, *SD*=1.21); *U*=2551, *z*=3.65, *p*<.001, *r* = 0.32. There were no significant differences between groups on the influence of who was running the study ( *U*=1869, *z*=-0.37, *p*=.71, *r*=0.03) (NDC *M*=4.4, SD=0.92, TD *M*=4.4, SD=0.99) or the study data security procedures ( *U*=2011, *z*=0.53, *p*=.6, *r*=0.05)(NDC *M*=4.41, SD=0.79, TD *M*=4.24, SD=1.02).

The influence of who was running the study on data sharing preferences was significantly affected by parental education [X ^2^(2)=7.348, *p=*.025, η2=.047]; those parents with an undergraduate or postgraduation education were more influenced by the organisation conducting the study in their decision to share their child’s data compared to those parents with a primary/secondary education (z = 2.576, *p*= .030 and z = 2.601, *p*= .028 respectively). There was no significant difference between parents with an undergraduate or postgraduate education on the influence of who is running the study on their willingness to share their child’s data (z= .109, *p*= .913). See Table S6 for mean ranks and standard deviations. Parental education did not affect the influence of the Purpose of the study [X ^2^(2)=.040, *p=*.980, η2=.0001] or the influence of data security procedures [X ^2^(2)=2.099, *p=*.350, η2=.015] on data sharing preferences.

 We also examined whether parental ethnicity affected the factors that parents rated as most influential in making the decision to share their child’s data. We found that the influence of who was running the study was affected by parental ethnicity [X ^2^(3)=8.321, *p=*.040, η2=.044]. Parents who were mixed race/other (M = 5.00, SD = .00) were more influenced by who was conducting the study in their decision to share their child’s data compared to those parents who were White (M= 4.35, SD= .984) (z= 2.672, *p*= .045). There were no significant differences between mixed race/other parents and Black (M= 4.40, SD= .548)(z= 1.952, *p*= .306); or Asian parents (M= 4.17, SD= 1.193)(z= 2.449, *p*= .086) in the influence of who’s running the study on data sharing. Additionally, there was no significant differences on the influence of who’s running the study between Black and Asian parents (z= .073, *p*= 1.00); Black and White parents (z= .487, *p*= 1.00) or Asian and White parents (z= .602, *p*= 1.00). See Table S7 for mean ranks and standard deviations.

### How can I control my child’s data?

Parents were introduced to four main types of consent with differing levels of control (Restrictive, Tiered, Broad and Dynamic; see SM1.3) with pros and cons of each. Parents were asked what type of consent they would be most comfortable with giving for their child’s data, what type of data release they would be happy with and how much they worry about their child’s wishes towards their data as they grew older. 

**Figure 4.**
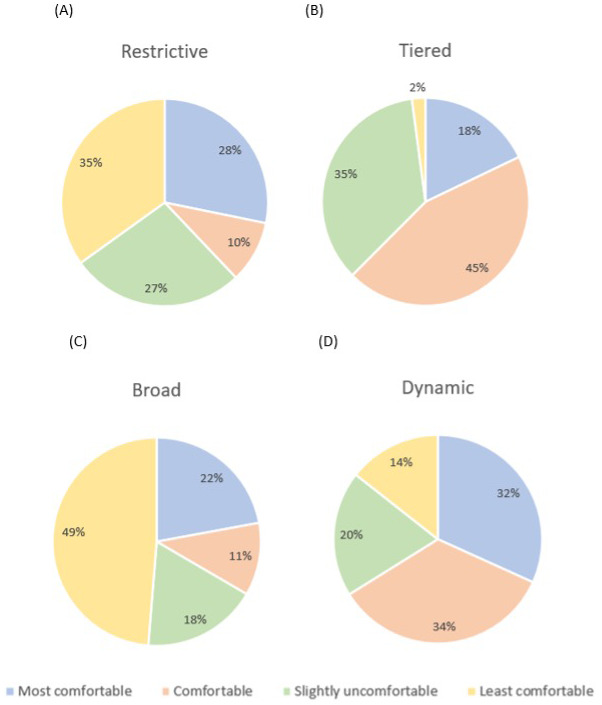
Graphs to show participants ranked preference of different consent models from most to least comfortable (Panel **A**: Restrictive, Panel **B**: Tiered, Panel **C**: Broad, Panel **D**: Dynamic).

***Overall consent preference.*** Participants were asked to rank the 4 levels of consent (Restrictive, Tiered, Broad and Dynamic) in order of most to least comfortable. A Friedman's analysis of variance was significant [χ ^2^(3)=47.75, *p*<.001, W=.08; Figure 3]. Follow-up Dunn’s post-hoc tests with Bonferroni adjustments (alpha corrected to *p*=.008) revealed significant differences between Dynamic and Restrictive consent (z=.51, *p*=.001), and Dynamic and Broad consent (z=.77, *p*<.001), with Dynamic consent consistently being rated as a more comfortable consent preference than the others. Similarly, Tiered consent was rated as a significantly more comfortable sharing option than Restrictive (z=.45. *p*=.003) and Broad consent (z=-.72, *p<*.001). There were no significant differences between the ratings of Dynamic vs Tiered consent (z=.05, *p*=1) or Restrictive vs Broad consent (z=-0.26, *p*=.27; see Figure 4). There are no significant group differences when comparing TD and NDC ratings of level of comfort with the four consent types (Table S1).

 We investigated whether parental education influenced participants ratings of the different consent models, no significant result was found; Restrictive (X ^2^(2)= 3.338, *p*= .188, η2=.019), Tiered (X ^2^(2)= .895, *p*= .639, η2=.006), Broad (X ^2^(2)= .683, *p*= .711, η2=.003) and Dynamic (X ^2^(2)= .955, *p*= .620, η2=.008). When examining whether parental ethnicity impacted participants’ overall consent preferences, we also found no significant differences [Restrictive: (X ^2^(3)= 1.890, *p*= .596, η2=.009); Tiered: (X ^2^(3)= 1.889, *p*= .596, η2=.014); Broad: (X ^2^(3)= .032, *p*= .998, η2=.0002) and Dynamic: (X ^2^(3)= .131, *p*= .988, η2=.002]. See Table S8 and S9 for mean ranks and standard deviations.

***Types of data release.*** Participants were asked what type of data release they would be happy with if their child’s data was anonymised and shared in a secure way. One-third (33%) of participants responded that they would be happy for this data to be shared in a public data release, while two-thirds (66.7%) indicated a preference for restricted data release. We found similar results when parents considered their own personal data (37.4% were comfortable with a public data release, whilst 62.6% preferred a more restrictive data release; McNemar’s test [χ ^2^(1)=2.72, *p*=.1, V=.8].

We found significant group differences in relation to data release types for child data [χ ^2^(1)=4.38, *p*=.036, V=.15], with TD families more likely to prefer restrictive data releases (TD restrictive=72.1%, TD public=27.9%) than NDC families (NDC restrictive=57.5%, NDC public=42.5%). There was no significant difference when parents were asked to consider which type of data release they would be most comfortable with when thinking about their own data [χ ^2^(1)=1.26, *p*=.262, V=.08], (TD restrictive=65.6%, TD public=34.4%, NDC restrictive=57.5%, NDC public=42.5%). Parental education level did not affect child [χ ^2^(2)=1.38, *p*=.502, V = .085] or parent data release preference [χ ^2^(2)=0.98, *p*=.614, V=.07]. Similarly parental ethnicity did not significantly affect child [χ ^2^(3)=3.107, *p*=.375, V=.127] or parent [χ ^2^(3)=2.664, *p*=.446, V=.117] data release preference.

***Worry about child's wishes.*** Participants were asked to imagine they had taken part in a study with their child when they were an infant and to rate how much they would worry about their child’s wishes towards their data as they grew older, using a Likert scale from 1 (Not at all) to 5 (Very much so). We considered the impact of prior research experience (e.g., if families who had taken part in any form of research differed in their views to those families that had no previous research experience). We found significant differences [χ ^2^(4)=10.19, *p*=.037, V=.23], with more families with no research experience (72.7%) ‘somewhat worrying’ about their child’s future wishes compared to those families with research experience (28.3%); [χ ^2^(1)=9.61, *p*=.001; alpha corrected to *p*=.005 for multiple comparisons]. There were no significant differences between families with and without neurodevelopmental conditions [ *U*=3994, *z*=-1.24, *p*=.217, *r*=.09]. Neither parental education [χ ^2^(2)=1.926, *p*=.382, η2= .010] or parental ethnicity [χ ^2^(3)=1.820, *p*=.610, η2= .015] had a significant effect on participants worry about their child’s wishes. See Table S10 and S11 for mean ranks and standard deviations.

### What am I sharing?

Participants were asked to consider different types of data (e.g., questionnaires or experimental measures) and how willing they were to complete and share each type of data. Participants were asked to rate this on a scale from 1 (would not complete) to 5 (happy for this data to be shared freely). This is particularly important to identify what types of data participants would be happy to complete/share in order to inform study design with respect to future data sharing.

***Biological data.*** A significant difference was found between the type of biological data and parents' willingness to complete and share this measure [Friedman’s χ ^2^(6)=265.15, *p*<.001, W=.23]. Follow up Dunn’s post-hoc tests with Bonferroni adjustments revealed parents were significantly less comfortable to share DNA than other biological measures e.g. hormone levels (z=-.86, *p*=.002), pregnancy biological data (z=-1.22, *p*<.001, brain scans e.g. MRI (z=1.26, *p*<.001), non-invasive brain imaging (z=1.54, *p*<.001), physiological data (z=1.65, *p*<.001) and eye-tracking (z=-1.66, *p*<.001). Follow up tests revealed parents were less comfortable sharing other biological data such as hormone levels than sharing non-invasive brain imaging (z=.67, *p*=.045), physiological data (z=.79, *p*=.007) and eye-tracking (z=.8, *p*=.006); see Table S2.

***Personal/demographic data.*** We found significant differences between the types of personal and demographic data parents were willing to share [χ ^2^(4)=32, p<.001, W=.04]. Parents were more willing to share parent demographics (mean rank=3.17), when compared to household information, such as first part of postcode (z=.46, *p*=.042; mean rank=2.71). This was also the case for parent substance use (mean rank=3.18), which was found to be more willingly shared when compared to household information (z=-.467, *p*=.036).

***Questionnaire data.*** We found significant differences in parents’ willingness to share questionnaire data [χ ^2^(5)=88.65, *p*<.001, W=.09]. Parents were more comfortable sharing child sleep data (mean rank=3.82) compared to sharing pregnancy questionnaires data (mean rank=3.24; z=.58, *p*=.032).

***Assessment data.*** We used a Wilcoxon signed-rank test to investigate if there were differences in parents' self-reported willingness to share clinical assessments (such as those for neurodevelopmental diagnostic purposes) compared to developmental assessments that measure whether children are meeting developmental milestones. Parents were more willing to share the outcome of developmental assessments (mean=3.78, SD=0.93) than clinical assessments (mean=3.69, SD=1.00; z=-3, *p*=.003, r=0.22).

***Group differences.*** We examined if there were differences between NDC and TD groups willingness to share different types of data. The NDC group were more willing to share brain scans [U(194)=5358, z=2.5, *p*=.012], DNA [U(194)=5619, z=3.16, *p*=.002], pregnancy biological data [U(194)=5375, z=2.53, *p*=.011], other biological data [U(194)=5346.5, z=2.45, *p*=.014], child demographics [U(194)=5401.5, z=2.6, *p*=.009], parent demographics [U(194)=5251.5, z=2.19, *p*=.028], clinical assessments [U(194)=5107, z=2.38, *p*=.017], family medical and psychiatric histories [U(194)=5219, z=2.1, *p*=.036], pregnancy questionnaires [U(194)=5286.5, z=2.29, *p*=.022] and parent substance use questionnaires [U(194)=5184, z=2, *p*=.046] compared to the TD group. We found no group differences in willingness to share other measures, such as parental mood and stress data (see Table S2 for a full list of measures).

**Figure 5.**
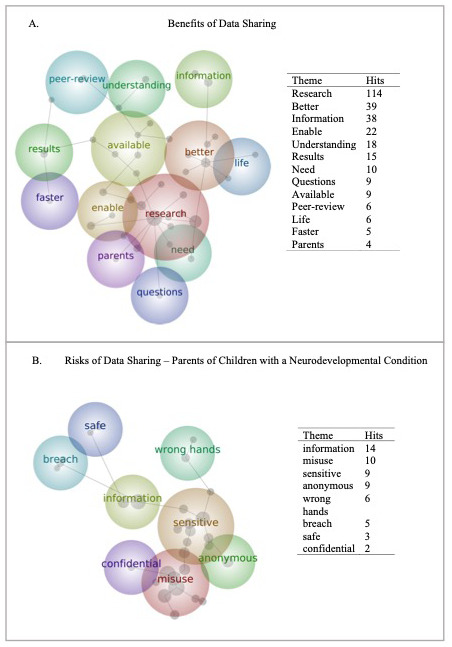
Word map of parents’ opinions on the Benefits (Panel **A**) and Risks (Panel **B**) of data sharing. Panel B is restricted to those parents with a child with one or more neurodevelopmental condition.

***Qualitative results.*** The questionnaire also consisted of four free text questions, with no character limit. Parents were given the opportunity to report on what they viewed as the main benefits and risks of sharing their own/their child’s data. We used Leximancer Desktop 5.0 ( www.leximancer.com) to generate concepts and themes based on responses to these questions. This analysis was not intended to be exhaustive, but was included to identify any concepts of importance to parents which might have been overlooked when designing the survey. We used Leximancer’s concept map tool to display topographically the prevalence and co-occurrence of concepts and themes from the qualitative data (see Figure 5).

Parents favoured themes like “better”, “research” and “understanding” when describing the benefits of data sharing, indicating that supporting the improvement of research quality was the primary benefit of data sharing. The proximity of the themes “results”, “available” and “faster”, indicate that parents felt that expediting the research process was another important benefit of data sharing.

Parents describing the risks of data sharing used terms like “information and “misuse”, both proximate to each other topographically and with high respective prevalence, indicating that the misuse of the information, or data, shared, was a key concern. Concerns about data “breaches” and data falling into the “wrong hands” were topographically proximate, though had fewer prevalence hits than other concepts. Parents of children with one or more neurodevelopmental condition were particularly concerned with the “sensitivity” of data being shared – this theme had high prevalence for this group *only,* and was proximal to other key concepts, particularly “misuse.”

## Discussion

Data sharing is an increasingly important focus for developmental science ( Friedman, 2007), but we need to move forward in partnership with parents and families. To establish family views on key parameters of data sharing, we conducted a comprehensive survey of the views of parents of young children. We assessed a heterogenous sample enriched for families with a history of neurodevelopmental conditions to establish whether motivations may differ based on experience with clinical services. We focused on views of sharing of pseudoanonymised data (with contact details removed but identified with an ID code, as is common in most developmental research), because personal data is already governed by strict privacy and security frameworks. Results showed that families preferred consent models that gave them maximal control over sharing their child’s data (dynamic or tiered consent). Further, only a third of families indicated that they were comfortable with completely open sharing, meaning that funders and journals that mandate open sharing will risk substantially skewed samples of enrolled participants; only 55% of families were happy with global data sharing, posing challenges for the globalisation of research collaborations. Trust in industry was particularly low, a consistent theme in other work that indicates the need for continued work in communicating the goals and values of industrial partners ( Manhas *et al.*, 2015). Importantly, the purpose of a research study was as important in decision-making as security and the nature of the people conducting it; this indicates families are thinking beyond their own child’s right to privacy and considering more altruistic aspects of societal research goals but also that efforts to improve data security are necessary but not sufficient for an ethical data sharing policy. In general, families with experience of neurodevelopmental conditions were happier to support data sharing than other families; this raises important considerations for data sharing dialogues that often focus on these communities but may assume that parents with typically developing children have fewer concerns. Taken together, developmental science requires databasing approaches that allow shared decision making and embed transparency about the purposes for which data is shared.

### Variation by location and sector

Trust and willingness to share data varied by key factors such as geographical location and sector. Overall, whilst 96% of parents said they would be happy to share their child’s data with universities and research centres, only 17% of participants would be happy to share their child's data with private companies and industry. This lack of trust in the private sector has been previously noted ( Manhas *et al.*, 2015) and indicates substantial barriers to collaborative projects that include industrial partnerships. Further work is required to determine the reasons for mistrust, but high-profile concerns around the approach of companies such as Cambridge Analytica ( Berghel, 2018) and mistrust of the profit motives of private companies and their contribution to (for example) the opioid crisis ( Marks, 2020) are likely contributory factors. Outreach events in which companies can discuss their approach to research with parents are important, as well as maintaining the highest standards of probity and transparency in industrial collaborations. The common failures to declare conflicts of interest in published studies or press releases undermine these efforts (e.g., Feldman & Mann, 2019).

Geographical location of a sharing partner was also relevant. Although 55% of this UK sample said they would be happy to share their data globally, 24% preferred their data to stay ‘Within the EU’ and 15% selected ‘UK only’ wide data sharing (with ‘None of the above’ at 6%). The democratisation of research data is a critical endeavour ( Buckingham *et al.*, 2012), with emphasis needed on both collecting data from contexts outside industrialised settings ( Nielsen *et al.*, 2017) but also sharing already-collected data with scientists from around the world. Data sharing is important for pooling insights across larger populations; several recent publications on linking individual differences in brain function to behaviour suggest sample sizes need to reach the hundreds of thousands to be meaningful ( Grady *et al.*, 2021), and in genetics samples in the millions will be required ( Hivert *et al.*, 2021). Such samples are rarely easy to reach without data pooling across settings, and this process is in its infancy for most developmental research. Thus, parental reticence about sharing beyond local geographical borders is a challenge to the globalisation of research. Some groups have managed this successfully. Consortia like the ‘Many Babies’ group (e.g., Visser *et al.*, 2022) play a critical role in deploying the same experiment across continents to assess reproducibility and generalisability; methods employed are typically visual attention or eyetracking (the methodology that raised the fewest concerns in our respondents). Databrary provides an innovative platform for video sharing ( http://databrary.org). Many behavioural cohorts have infant data with permission for broad sharing. However, databases that include multiple linked datapoints per participant with brain and biological data from infancy that can be openly shared remain rare. Further, there is very little empirical literature on the experience of parents and children in these studies, their decision–making around data sharing and their understanding of the open nature of the data. 

### Family experience of neurodevelopmental conditions

Families with a child or family member with a neurodevelopmental condition (primarily autism or ADHD) were in general more positive about data sharing than families who only had experience of typically developing children. Families with experience of neurodevelopmental conditions may have a stronger motivation to share data in order to help their child or other children experiencing similar challenges (e.g., Haas *et al.*, 2016; Lajonchere, 2010; Salvaterra *et al.*, 2014). Care must be taken not to exploit this altruistic motivation and to ensure that data sharing protections are just as strong for this group. In particular, attitudes of autistic people and family members of autistic people can sometimes diverge; ensuring that infants or children have control over their own data as they grow old enough to make decisions for themselves is critically important. Since parents are making decisions about sharing on behalf of their child, consideration of the child’s future self is important. For example, discoveries about a child’s genetics can have far-reaching implications for them having children in the future. For this reason, general NHS practice is not to test for genetic syndromes in children who are not “Gillick competent” (able to consent) unless they are immediately consequential for treatment ( British Society for Human Genetics, 2010; Joint Committee on Genomics in Medicine, 2019); these norms are beginning to shift in other countries and settings ( Papaz *et al.*, 2019) and raises important questions about the ethics of returning genetic results from a study where data is collected at multiple global sites. In the case of neurodevelopmental conditions such as autism there are substantial concerns around the development of prenatal tests that could lead to selective abortion ( Walsh *et al.*, 2011); this is more likely in the case of rare variant detection given their larger effect sizes on developmental outcomes. Open sharing of a child’s data including their genetic information cannot be retrieved; many autistic people have substantial concerns about genetic data sharing ( Sanderson, 2021) and it is unclear how knowing their DNA was shared as a child could affect them. Studies exploring the feelings of adults whose data was shared as a child and of teenagers ( Pavarini *et al.*, 2022) are important in this regard. 

### Research goals

Families are concerned not only about security and privacy of their child’s data, but the purpose for which it is shared. These concerns are greatest in families with personal experience of neurodevelopmental conditions. Conversations about the goals of research have become increasingly prevalent on social media and in research collaborations, with the neurodiversity movement at the cutting-edge of highlighting the common mismatches between a study’s stated scientific goals and their public communication (e.g., Pellicano *et al.*, 2014). Increasing awareness of the bias that can be introduced by ableist language ( Bottema-Beutel *et al.*, 2021) and deficit-based models ( Kapp, 2019) has led to an increasing shift in thinking and approach in many developmental fields ( Pellicano & den Houting, 2022). Together, there is a growing understanding that families are not simply concerned with the implications for their own child of a data sharing breach (e.g. the loss of their right to an open future through inflicted insight, or the loss of privacy if information is reidentified). Rather, families are concerned that their child’s data might be used for a study with a purpose with which they disagree. An area of common concern are studies with the goal of identifying prenatal tests for neurodevelopmental conditions (known to enable abortion in the case of Down’s syndrome; Natoli *et al.*, 2012). High profile instances of similar issues have been widely discussed in the case of cultural groups and DNA data (e.g., Lee *et al.*, 2019) and the use of cell lines without permission ( Wolinetz & Collins, 2020). We need to carefully consider mechanisms that could allow families to decide against data sharing with studies with a particular goal. One approach (the most burdensome) is to ask families to reconsent to each new episode of data sharing, but this requires data to remain identifiable so that it can be shared (or not shared) on each new occasion. This trade-off of identifiability and control must be carefully considered at study outset, but notably dynamic consent was the clear preference expressed in our survey. A less onerous method may be to develop broad categories of purpose that families could agree or disagree with during the original consenting process; online portals can then allow these preferences to be changed at will by the parent or by the child as they grow (then frozen when delinkage occurs). Alternatively, participants could apply to join a panel that would be responsible for collective decision-making about acceptable purposes on behalf of the cohort (allowing deidentification to occur). Applicants to access study data can then describe the purpose to which the data will be put. By essentially requiring a preregistration, this approach can also slow the process of dataset decay ( Thompson *et al.*, 2020) and constrain statistical flexibility, allowing Type 2 error rates to be tracked. This curated sharing approach thus provides an attractive balance between open science and privacy and protection, but requires considerable investment by the scientific community in scientific review.

### Biased samples

More personalised approaches to curated data sharing raise substantial analytical issues. Given only 33% of our parents said they would be happy with open data sharing, investigators who mandate sharing risk attracting a biased sample of families to the research project. This may exacerbate inequalities in the representation of minoritized groups in research studies. The overwhelmingly White/educated/industrialised nature of research samples in developmental science has been well documented ( Nielsen *et al.*, 2017); efforts to improve this need to be considered alongside initiatives to increase possibilities for open data sharing. Rigorous evaluation of the demographic characteristics of participants who have different levels of willingness to share data is important in this endeavour, along with increasing trust in the research process in historically marginalised communities. Comfort with sharing did vary slightly between modalities, with the most concerns for sharing DNA data and the fewest for eyetracking data. However, the differences were small and in general the data indicated that researchers need to consider family concerns for all collected data types. Additionally, we found no differences in parental level of education and ethnicity between those parents that are willing to share their child’s data and those parents that would prefer to restrict their child’s data. As such, the sample of parents that are willing to share their child’s data seem to be representative on these core characteristics. This may lend support for the suggestion that “the claims of the amount of consent bias are likely overstated; and any residual effects of consent bias fall below acceptable levels of imprecision” ( Rothstein & Shoben, 2013).

Including a broad range of participants in a research study with tiered or dynamic permissions for sharing also poses challenges for reproducibility. If data shared with a group on a different continent contains a different number of participants than data shared with a local group, assessing the reproducibility of a research finding is challenging. One approach is to simulate missing data so that its statistical characteristics are preserved (e.g., Woods *et al.*, 2021); though it is important to have a full ethical discussion about whether this is in the spirit of the participant’s decision to restrict the sharing of their data. Imputation approaches should become standard in data repositories such that this can be reproducibly achieved in a standardised way that can be described in publications resulting from shared data. However, many missing data approaches may be unsuitable if data is not missing at random ( Little *et al.*, 2014); again, this highlights the need to first understand the characteristics of the individuals who do and do not choose to share data openly. Further qualitative study of the reasoning behind an individual’s choice to restrict sharing may also open avenues – if concerns are primarily around privacy, federated data sharing approaches or full information about security and privacy considerations may enable them to feel confident about broader sharing. 

### Limitations

Though we attempted to include a broad sample in the current investigation, we are aware that much of the sample is from a White British background (74%) with higher familial education levels (~88% with an undergraduate degree). Also, the study was conducted within a UK context only. As such, opinions from different contexts and cultures are important to consider.

In addition to this, a large proportion of our sample had previous experience of participating in research (either themselves or their children). Whilst this is a strength in that participants are drawing on lived experiences to respond to the questions and not answering hypothetically, it is important to note that we may be overestimating the willingness to share data by virtue of not capturing a higher proportion of those respondents that may not participate in research at all. 

## Conclusion

Families want us to share their child’s data, but to do it very carefully. We need a field-wide investment in data sharing architectures that enable dynamic or tiered consent, implement state-of-the-art approaches to dealing with missing data, and embed full transparency to build community trust in the security, privacy and goals of different groups of researchers. We need a stronger emphasis on responsible open science, twinning the important drive for greater data democracy with an understanding of participant concerns and a commitment to ensuring that datasets do not become even less representative because of data sharing efforts. Taken together, we need to work in partnership with families to produce data sharing architectures that enables developmental science to become truly global.

## Data Availability

OSF: Parent attitudes towards data sharing in developmental science. https://osf.io/kajcg ( Begum, 2023) This project contains the following underlying data: Parent attitudes to data sharing.sav This project contains the following extended data: Holman, Begum-Ali et al. Parent attitudes to data sharing Supplementary Materials.docx Data are available under the terms of the Creative Commons Attribution 4.0 International license (CC-BY 4.0).
