# Supplementary material for: Parent attitudes towards data sharing in developmental science
Source: Open Res Eur. 2024 Jun 3;3:182. Originally published 2023 Oct 20. [Version 2] doi: 10.12688/openreseurope.16516.2 (PMC11245672; doi:10.12688/openreseurope.16516.2)
Supplement: Peer review history

# Reviewer report: Reviewer response for version 2

**DOI:** 10.21956/openreseurope.19123.r41182

**Published:** 2024-07-12

**Authors:** Silvia Rigato

**Affiliations:** 1 Department of Psychology, University of Essex, Colchester, England, UK

I have no further comments.

If applicable, is the statistical analysis and its interpretation appropriate?

Yes

Is the study design appropriate and is the work technically sound?

Yes

Is the work clearly and accurately presented and does it engage with the current literature?

Yes

Are the conclusions drawn adequately supported by the results?

Yes

Are sufficient details of methods and analysis provided to allow replication by others?

Yes

Are all the source data and materials underlying the results available?

Yes

Reviewer Expertise:

Developmental science

I confirm that I have read this submission and believe that I have an appropriate level of expertise to confirm that it is of an acceptable scientific standard.

# Reviewer report: Reviewer response for version 2

**DOI:** 10.21956/openreseurope.19123.r41183

**Published:** 2024-06-04

**Authors:** Evelyne Mercure

**Affiliations:** 1 Department of Psychology, Goldsmiths University of London, London, England, UK

The authors have fully addressed my previous comments.

If applicable, is the statistical analysis and its interpretation appropriate?

Yes

Is the study design appropriate and is the work technically sound?

Yes

Is the work clearly and accurately presented and does it engage with the current literature?

Yes

Are the conclusions drawn adequately supported by the results?

Yes

Are sufficient details of methods and analysis provided to allow replication by others?

Yes

Are all the source data and materials underlying the results available?

Yes

Reviewer Expertise:

Developmental cognitive neuroscience

I confirm that I have read this submission and believe that I have an appropriate level of expertise to confirm that it is of an acceptable scientific standard.

# Reviewer report: Reviewer response for version 1

**DOI:** 10.21956/openreseurope.17830.r35777

**Published:** 2023-11-20

**Authors:** Evelyne Mercure

**Affiliations:** 1 Department of Psychology, Goldsmiths University of London, London, England, UK

This article investigates parental opinions on data sharing in developmental science. This is an important and rarely addressed topic with clear implications for funders’ and publishers’ data sharing policy. Data is presented from 195 parents who responded to a survey designed by the authors. The data suggests that parents are generally supportive of curated data sharing, with increased willingness to share their child’s data with universities, research centres, GPs and hospitals than private companies and industry. Parents supported more nuanced consenting models with opportunities to modify their permission over time. Parents with family experience of neurodevelopmental conditions were generally more open to data sharing than families without experience of neurodevelopmental conditions. The article is well-documented and written and presents a study which is robustly designed, conducted, and analysed. I have a few suggestions for clarifications and improvements.

 The different types of consent could be more clearly explained in the introduction and/or method section. It is difficult to fully understand this section of the results as it currently is. In the discussion, different consent scenarios are described, but it is not always straightforward to match these to the labels that are used in the questionnaire and the results section.

 p.3: The authors state that ‘there has been little focus on the gender distribution of researchers who exploit open datasets’. Could the authors clarify why this gender distribution is of interest?

 Figure 2.: The colour scale seems to be missing from the bottom panel.

 Figure 3: The question presented on the bottom panel does not seem to match the colour scale.

 In personal/demographic data, it is not always clear what information would be classified as personal and demographic versus household information. I also wondered how this was presented to parents and if all parents would have understood these terms in a similar way.

 The same goes for questionnaire data. What is this exactly and how was it described for parents?

 To interpret Figure 5, it would be helpful to know how many parents provided written responses. For example, when stating that a term had 114 hits, does this mean that it was presented in 114 out of 195 written reports?

If applicable, is the statistical analysis and its interpretation appropriate?

Yes

Is the study design appropriate and is the work technically sound?

Yes

Is the work clearly and accurately presented and does it engage with the current literature?

Yes

Are the conclusions drawn adequately supported by the results?

Yes

Are sufficient details of methods and analysis provided to allow replication by others?

Yes

Are all the source data and materials underlying the results available?

Yes

Reviewer Expertise:

Developmental cognitive neuroscience

I confirm that I have read this submission and believe that I have an appropriate level of expertise to confirm that it is of an acceptable scientific standard, however I have significant reservations, as outlined above.

# Response

**Published:** 2024-04-23

**Authors:** Jannath Begum Ali

**Affiliations:** Department of Psychological Sciences, Birkbeck University of London, London, England, UK

**Reviewer 2 comments: **   This article investigates parental opinions on data sharing in developmental science. This is an important and rarely addressed topic with clear implications for funders’ and publishers’ data sharing policy. Data is presented from 195 parents who responded to a survey designed by the authors. The data suggests that parents are generally supportive of curated data sharing, with increased willingness to share their child’s data with universities, research centres, GPs and hospitals than private companies and industry. Parents supported more nuanced consenting models with opportunities to modify their permission over time. Parents with family experience of neurodevelopmental conditions were generally more open to data sharing than families without experience of neurodevelopmental conditions. The article is well-documented and written and presents a study which is robustly designed, conducted, and analysed. I have a few suggestions for clarifications and improvements.  

*We thank the Reviewer for their positive comments on our manuscript.*

 R2.1: The different types of consent could be more clearly explained in the introduction and/or method section. It is difficult to fully understand this section of the results as it currently is. In the discussion, different consent scenarios are described, but it is not always straightforward to match these to the labels that are used in the questionnaire and the results section.

*We have provided further details about the consent models (pg. 7):“How is the data shared? We asked participants about their views of common consenting models, including  Restrictive (participants are contacted and asked each time a study wants to use their previously collected data), Dynamic (participants use an online portal to choose which studies their data is shared with), Tiered (participants choose certain categories/tiers of research they would be happy to share their data with at the point of informed consent for the original data collection) and Broad consent (the data can be shared in an anonymised manner for other studies and participants are not contacted for permission).”*

 R2.2: p.3: The authors state that ‘there has been little focus on the gender distribution of researchers who exploit open datasets’. Could the authors clarify why this gender distribution is of interest?

*As per R1.2, we have further expanded upon this point (pg. 4):* *“Further, there has been little focus on the gender distribution of researchers who exploit open datasets (sometimes called ‘data parasites’; Longo & Drazen, 2016) relative to those who often are involved in preparing them. This can have real implications for the career development of researchers who collect and share data (particularly if they are at an earlier career stage) because this process requires a considerable investment of time relative to running analyses on existing data. Specifically, it has been found that female academics are assigned and complete more ‘academic housework’, such as mentoring, student and faculty service (including emotional labour) and being involved in lower status committees that do not necessarily get reflected in their CVs (O’Meara et al., 2017; Hanosono et al., 2019; Jarvinen & Mik-Meyer, 2024). Indeed, this ‘invisible labour’ could also be extended to the time intensive tasks of data collection and curation that are specific to open datasets, with women typically overrepresented in more junior (e.g., data collection) roles, but underrepresented in more senior academic roles (Herschberg and Berger, 2015). In order for open datasets to avoid becoming a route where gender disparities are amplified, it would be important to examine the mechanisms of this unequitable gender distribution and remedy this.”*

 R2.3: Figure 2.: The colour scale seems to be missing from the bottom panel.

*We have now added the legend for Figure 2 (pg. 40)*

 R2.4: Figure 3: The question presented on the bottom panel does not seem to match the colour scale.

*We have now corrected this to “How much would you worry about your child’s wishes towards their data as they grew older?” (pg. 41).*

 R2.5: In personal/demographic data, it is not always clear what information would be classified as personal and demographic versus household information. I also wondered how this was presented to parents and if all parents would have understood these terms in a similar way.

 The same goes for questionnaire data. What is this exactly and how was it described for parents?

*Given the close nature of these types of information, we agree that it may have been confusing for the parents. As such, in the questionnaire (detailed in the SM), we defined the exact types of information for each category. For example, we asked specifically “How happy would you be to share demographic information about your child, including ethnicity and date of birth?”, “How happy would you be to share demographic information about yourself, including ethnicity, date of birth and education level?” and “How happy would you be to share information about your household, including number of bedrooms, first part of postcode and area?”* *We also provided several, separate options regarding different types of questionnaire data. Specifically, we asked: “Please select all the types of questionnaires you would be happy to be shared:* *Child’s sleep patterns,* *Child’s temperament (personality),* *Child’s milestones (e.g., when did they start crawling?),* *Parent(s) mood (e.g., questions about symptoms of depression),* *Parent(s) stress levels,* *Family medical and psychiatric history,* *Pregnancy data (e.g., gestation, any medications taken during pregnancy),* *Parent(s) substance use.”* *We have also clarified this in the main text (pp. 8-9):* *“Section 1 was designed to collect basic demographic information about their youngest child (sex, age, ethnicity) and family (highest level of education of the parent filling in the questionnaire, presence of neurodevelopmental or genetic conditions).”* *“Personal/Demographic data* *We found significant differences between the types of personal and demographic data parents were willing to share [χ ^2^(4)=32,* *p<* *.001, W=.04]. Parents were more willing to share parent demographics such as ethnicity and education level (mean rank=3.17), when compared to Household information, such as the first part of postcode or the number of bedrooms in the home* *(z=.46, p=.042; mean rank=2.71). This was also the case for parent substance use (mean rank=3.18), which was found to be more willingly shared when compared to Household information (z=-.467, p=.036).” (pp. 18-19).* *As such, given the detailed information for each type of data, we feel that this would have been fairly clear to participants.*

 R2.6: To interpret Figure 5, it would be helpful to know how many parents provided written responses. For example, when stating that a term had 114 hits, does this mean that it was presented in 114 out of 195 written reports? *Our final sample completed the questionnaire in full (N = 195), which included the free text responses regarding the benefits and risks of data sharing. We have made this clear in the Figure captions (pg. 38):* *“Figure 5: Word map of all parents’ (n = 195) opinions on the Benefits (Panel A) and Risks (Panel B) of data sharing. Panel B is restricted to those parents with a child with one or more neurodevelopmental condition (n = 73).”*

# Reviewer report: Reviewer response for version 1

**DOI:** 10.21956/openreseurope.17830.r35776

**Published:** 2023-11-10

**Authors:** Silvia Rigato

**Affiliations:** 1 Department of Psychology, University of Essex, Colchester, England, UK

In this work, the authors investigated parents' views on data sharing in developmental science. Given the current situation of funders, publishers and institutions increasingly encouraging open data access, this is really timely. I read this manuscript with great interest and I agree with the authors that the question of data sharing is an important one, for both researchers and parents. The authors conducted a survey online and report data from 195 parents of children with and without neurodevelopment disorders. I believe the methods and the analyses employed are valid and the results will be of interest not only to the scientific community and those taking part in research but also to a range of stakeholders. The discussion provides informative conclusions and offers thoughtful reflections. The authors also advance possible directions in the area of data sharing.

 I outline below some minor comments and suggestions:

1. The first part of the second paragraph reads a bit redundant with the introductory paragraph. 
2. I find it interesting the reflection on gender distribution of researchers who prepare and exploit open datasets but I believe it needs a slightly bigger context or explanation to be fully appreciated by the reader.
3. Please specify whether the information of parent education refers only to the parent filling in the survey.
4. Figure 1: I think the arrow to the box  'No questions completed (n=105)' should come from the box 'Completed consent form (n=300)'.
5. Figure 2: missing legend for graphs (E) (F) (G).
6. Do the authors think the answers to the questions on the types of organisations (who can share the data and trust in these organisations) would have been different if the survey was coming from a different organisation, i.e. private company, charity, GP? Consider to comment on this point in the discussion. 

If applicable, is the statistical analysis and its interpretation appropriate?

Yes

Is the study design appropriate and is the work technically sound?

Yes

Is the work clearly and accurately presented and does it engage with the current literature?

Yes

Are the conclusions drawn adequately supported by the results?

Yes

Are sufficient details of methods and analysis provided to allow replication by others?

Yes

Are all the source data and materials underlying the results available?

Yes

Reviewer Expertise:

Developmental science

I confirm that I have read this submission and believe that I have an appropriate level of expertise to confirm that it is of an acceptable scientific standard, however I have significant reservations, as outlined above.

# Response

**Published:** 2024-04-23

**Authors:** Jannath Begum Ali

**Affiliations:** Department of Psychological Sciences, Birkbeck University of London, London, England, UK

**Reviewer 1 comments:**   In this work, the authors investigated parents' views on data sharing in developmental science. Given the current situation of funders, publishers and institutions increasingly encouraging open data access, this is really timely. I read this manuscript with great interest and I agree with the authors that the question of data sharing is an important one, for both researchers and parents. The authors conducted a survey online and report data from 195 parents of children with and without neurodevelopment disorders. I believe the methods and the analyses employed are valid and the results will be of interest not only to the scientific community and those taking part in research but also to a range of stakeholders. The discussion provides informative conclusions and offers thoughtful reflections. The authors also advance possible directions in the area of data sharing.

*We thank the Reviewer for their positive assessment of the study.*

 R1.1: I outline below some minor comments and suggestions:The first part of the second paragraph reads a bit redundant with the introductory paragraph.   

*We have removed this portion of the second paragraph (pg. 3) *  

 R1.2: I find it interesting the reflection on gender distribution of researchers who prepare and exploit open datasets but I believe it needs a slightly bigger context or explanation to be fully appreciated by the reader.  

*We thank the Reviewer for this comment and have expanded our discussion of the gender disparity in open datasets (pg. 4):* *“Further, there has been little focus on the gender distribution of researchers who exploit open datasets (sometimes called ‘data parasites’; Longo & Drazen, 2016) relative to those who often are involved in preparing them. This can have real implications for the career development of researchers who collect and share data (particularly if they are at an earlier career stage) because this process requires a considerable investment of time relative to running analyses on existing data. Specifically, it has been found that female academics are assigned and complete more ‘academic housework’, such as mentoring, student and faculty service (including emotional labour) and being involved in lower status committees that do not necessarily get reflected in their CVs (O’Meara et al., 2017; Hanosono et al., 2019; Jarvinen & Mik-Meyer, 2024). Indeed, this ‘invisible labour’ could also be extended to the time intensive tasks of data collection and curation that are specific to open datasets, with women typically overrepresented in more junior (e.g., data collection) roles, but underrepresented in more senior academic roles (Herschberg and Berger, 2015). In order for open datasets to avoid becoming a route where gender disparities are amplified, it would be important to examine the mechanisms of this unequitable gender distribution and remedy this.”*  

 R1.3: Please specify whether the information of parent education refers only to the parent filling in the survey.  

*We have now clarified this with the following text (pp. 8-9): “Section 1 was designed to collect basic demographic information about their youngest child (sex, age, ethnicity) and family (highest level of education of the parent filling in the questionnaire, presence of neurodevelopmental or genetic conditions.”*  

 R1.4: Figure 1: I think the arrow to the box  'No questions completed (n=105)' should come from the box 'Completed consent form (n=300)'.   

*We have amended this in Figure 1 (pg. 39).*   R1.5: Figure 2: missing legend for graphs (E) (F) (G). *We have now added the legend for Figure 2 (pg. 40)*  

 R1.6: Do the authors think the answers to the questions on the types of organisations (who can share the data and trust in these organisations) would have been different if the survey was coming from a different organisation, i.e. private company, charity, GP? Consider to comment on this point in the discussion.   

*This is an interesting point and we have added the following text to the Discussion (pg. 23): “One point of note to the question of trust in organisations is that parental ratings may well have been influenced by the organisation conducting the research. For example, it is possible that if parents are willing to take part in research for certain organisations (in this case, completing questionnaires for a university), they may also be more willing to share their/their child’s data with the same type of organisations. As such, it may be that we are observing a possible overestimation of trust in universities and research centres by virtue of the sample that completed the questionnaire. Though, importantly, parents reported similar levels of trust in GPs and hospitals as that of universities (25% vs 32% of parents that trusted the respective organisations completely with their child’s data).”*
